# High beverage sugar as well as high animal protein intake at infancy may increase overweight risk at 8 years: a prospective longitudinal pilot study

**DOI:** 10.1186/1475-2891-10-95

**Published:** 2011-09-23

**Authors:** Peter JM Weijs, Laura M Kool, Nicolien M van Baar, Saskia C van der Zee

**Affiliations:** 1 Department of Nutrition and Dietetics, School of Sports and Nutrition, Hogeschool van Amsterdam, University of Applied Sciences, Amsterdam, The Netherlands; 2 Department of Nutrition and Dietetics, Internal Medicine, VU University Medical Center, Amsterdam, The Netherlands; 3 EMGO+ Institute for Health and Care Research, VU University Medical Center, Amsterdam, The Netherlands; 4 Department of Environmental Health, Public Health Service Amsterdam, Amsterdam, The Netherlands (address: Nieuwe Achtergracht 100, 1018 WT Amsterdam

## Abstract

**Background:**

Combined effects of early exposure to beverage sugar and animal protein and later life overweight risk have not been studied.

**Methods:**

A prospective longitudinal study was initiated in 2001 with 226 infants between 4 and 13 months of age. Dietary intake was assessed with a 2 day food record. Also information on infant body weight and socio-economic status was obtained at baseline. At 8 year follow-up in 2009, children were surveyed again. Main outcome measure was overweight at 8 years as defined by BMIsds > = +1.0. Also maternal BMI, present dietary intake and physical activity, were obtained by questionnaire and 2-day food record.

**Results:**

At the 8 year follow up, 120 children (53%) were surveyed again. Of those, questionnaires and food records were completed for 63 children, for the other 57 children only weight and height at 8 years was available; 20 out of 120 children (17%) were self-reported overweight at 8 years of age. Unadjusted odds ratios (ORs; 95% CI) for overweight at 8 years were 1.10 (1.02, 1.18) for beverage sugar intake per one percent of energy intake and 4.06 (1.50, 11.00) for the highest tertile of animal protein intake at infancy compared to the lowest two tertiles. After adjustment for sex, age, infant weight, breastfed at intake assessment, and socio-economic status, odds ratios were 1.13 (1.03, 1.24) for beverage sugar, and 9.67 (2.56, 36.53) for highest tertile of animal protein intake. In the subgroup with completed questionnaire (n = 63) ORs were also adjusted for current maternal overweight, more than 2 months full breastfeeding, physical activity, and energy intake, but ORs remained significantly associated with overweight at 8 years.

**Conclusions:**

A high intake of sugar containing beverages as well as animal protein in the first year of life may increase the risk of overweight at 8 years. The results of this pilot investigation should be confirmed in a larger cohort.

## Introduction

Obesity and childhood obesity are increasing and etiology, epidemiology and treatment should be adressed [1]. After fetal nutritional experiences and early breastfeeding in the first months of life, the introduction of complementary feeding along with either breastfeeding or formula feeding is important for long-term infant development [2-4]. A high infant protein intake has been suggested to be related to childhood overweight [5-8], although not confirmed in hispanic children [9]. The suggested mechanism is an accelerated growth during infancy via stimulation of insulin and IGF-1 [2]. Sugar containing beverage intake in childhood has been shown to be related to overweight in childhood [10], and this effect seems to extend into adulthood [11]. The suggested mechanism is that higher sugar containing beverage intake is not compensated for by lower energy intake from other sources [12]. More specifically it is suggested that beverages from fruit sources or corn syrup contain a high level of fructose [12,13]. Fructose does not increase insulin levels after intake as glucose does, which results in relatively high levels of plasma triglycerides as a result of unsuppressed lipolysis [13]. However, there is no conclusive evidence for fructose as the central cause of obesity [14,15]. Also very early postnatal high carbohydrate complementary feeding is suggested to be related to later overweight [3]. These effects might imprint metabolic changes that last into later years of life and predispose for obesity and cardiovascular illness [2].

That other factors play a role in the etiology of overweight is undoubted, including overweight parents and inactive lifestyle of child [16-18]. However, we were able to retrieve only one study on beverage sugar intake during infancy and risk of overweight development [19]. The European Society of Pediatric Gastroenterology, Hepatology, and Nutrition (ESPGHAN) states that there are few data on the effects of specific complementary foods on growth [20].

The aim of the present pilot study was to determine whether high beverage sugar intake or high animal protein intake during infancy is associated with an increased risk of overweight at the age of 8 years.

## Methods

### Subjects

In April 2001 226 parents of infants 4-13 months of age responded to a notice distributed through a publisher of a magazine aimed at parents of very young children. Almost equal samples were obtained at 5 different age groups (4, 6, 8, 10, and 12 months of age) to cover the larger part of infancy that is not exclusively based on breastfeeding and/or formula feeding. Parents/caretakers of infants were invited to complete a questionnaire on weight, height, and parental education. They also received a 2 day food record with instructions. Description of calculation of nutrient intake have been published before [21,22]. Parents/caretakers signed an informed consent form. All procedures were in accordance with the ethical standards of the Hogeschool van Amsterdam.

Five of the 226 parents were excluded because they had not explicitly provided permission to be approached again after the initial assessment. Therefore, in april 2009, 221 of the 226 parents/caretakers were mailed again with a letter of invitation, a short questionnaire and a 2 day food record. To the parents no direct reference was made to the problem of overweight or obesity, to avoid bias in the provided information. The response was 63 out of 221 complete questionnaires including the 2 day food record. The non-responders were approached again by telephone with only the question for current weight and height of their child. In this way another 57 children were included into this study. Total loss of follow-up (221-120 = 101) was due to migration (49 letters returned to sender) or failure to trace (52 no contact by telephone).

Socio-economic status was judged by parental education. Mothers education was devided into high and low level of education, with high level of education defined as university, higher professional education, or preparatory education (college) for scientific education. All other education was defined as low level of education.

### Dietary intake at infancy

By means of a 2 day food record (1 weekday and 1 weekend day) the parents provided information on actual consumption of food in portions, or weighed if necessary. Parents were specifically asked to subtract spilled or not consumed amounts. Portion sizes were translated into weight by standard portion sizes used in The Netherlands [23]. Food weights were then multiplied by actual contents of nutrients per 100 g food by using the Dutch Food Composition Table [24,25]. For foods that could not be translated to existing Dutch Food Composition Table codes, mostly meals in a jar, the nutrients were calculated using data on the nutrient levels of ingredients.

Sugar containing beverages were any drinks provided to the infant that contained mono- and/or disaccharides, thus both naturally sweetened, sweetened by industry or sweetened at home; however milk, milk products, mothers milk, and infant formula were excluded. Animal protein was defined as protein minus vegetable protein, which are both available from the Dutch Food Composition Table.

### Questionnaire at 8 years

In 2009 parents were asked for weight and height of father, mother and the 8 year old child. Parents were retrospectively asked for breastfeeding and formula feeding practices apart from the at baseline assessment of dietary intake. This retrospective information was complementary to the prospective information from dietary assessment at infancy. Parents were asked whether their child was ever breastfed or not, formula fed or not, a combination of breastfeeding and formula feeding, and for how long. The 2 day food record was calculated for current nutritional intake, in order to adjust for current energy intake. Parents were asked for total hours per week that the child currently spends on physical activity (4 items: sports at school, sports at club, free outside play, and active travel to and from school) and inactivity (3 items: television watching, computer use, and gaming).

### Definition of overweight

Current weight and height were self-reported. Weight and height were used to calculate body mass index (kg/m^2^). Since BMI is sex and age specific for children, the BMI standard deviation score (BMIsds) was used, as calculated with the Growth Analyzer http://www.growthanalyser.org. WHO BMIsds cut-off point of +1 and +2 was used to define overweight and obesity, equivalent to BMI 25 and 30 kg/m^2 ^at 19 years (http://www.who.int/growthref/who2007_bmi_for_age/en/index.html at 19-06-2009). Overweight at 8 years includes children with obesity in the present analysis. Current maternal overweight was defined as BMI > 25.

### Statistical analyses

Differences in continuous variables between infant age groups were assessed with an independent samples t-test or analysis of variance (ANOVA) and dichotomous variables with the Chi square test.

The relation between animal protein and outcome was nonlinear and therefore included as tertile in the model (highest tertile = 1, lower two tertiles = 0). Tertiles for animal protein intake were generated per age group (4, 6, 8, 10, 12 months), since protein intake at 4 months is very different from protein intake at 12 months. Beverage sugar intake was used as continuous variable. Analyses were performed on dichotomous outcome overweight at 8 years with logistic regression analysis. We performed separate analyses for the group with information about weight and height at 8 years (n = 120) and the subgroup with complete questionnaires at 8 years (n = 63). Model 1 is the unadjusted effect of beverage sugar or animal protein intake, both as percentage of energy intake. Model 2a and 2b are adjusted for sex, infant age, body weight, and socio-economic status based on highest level of maternal education [2]. Since animal protein intake for breastfed children is different from non-breastfed children, we also adjusted for breastfeeding at the time of intake assessment. Model 2 is adjusted for each other, thus both beverage sugar and animal protein. Model 3 is adjusted for both beverage sugar and animal protein, and for adjustments from model 2a/2b. Model 4 is also adjusted for confounders at age 8 years, namely current maternal overweight, current physical activity in hours per week, and current energy intake in kcal per day, as well as duration of full breastfeeding of more than two months [16].

In addition, linear regression analysis was performed with BMIsds as dependent variable and beverage sugar and animal protein intake as independent variable to quantify the possible effect on BMIsds.

Statistical analysis was performed by SPSS 17 (SPSS Inc. Chicago, USA), and a *P*-value less than 0.05 was considered statistically significant. Means in the text are presented with SD.

## Results

A total of 120 (64 boys, 56 girls) infant BMI were used for analysis. Mean infant (sd) ages of responders at the time of completing the questionaire were 4.8 (0.3), 6.9 (0.3), 8.8 (0.3), 10.9 (0.3), and 12.4 (0.2) months (see also Additional file 1, Table S1). Mean age at infancy was 8.7 (2.8; range 4.4-13.0) months and at follow-up 8.7 (0.3; range 8.3-9.1) years. Mean BMI and BMIsds at 8 years was 16.3 (2.1) and -0.12 (1.17). For the whole group with complete data at 8 years (n = 63) 48 (80%) of the children had ever been breastfed, and 22 (37%) still received breastfeeding at the age of 6 months. Further infant characteristics are presented in Table 1. Table 2 shows food, energy, protein, and sugar intakes in infancy for children being overweight or not at age 8 years [26]. Both beverage sugar and animal protein intake from complementary foods like meat, milk and meals from a jar were higher in overweight children when adjusted for total energy intake. Overweight children consumed more drinks and less infant formula, and therefore also less protein from formula. The intake of beverage sugar and animal protein adjusted for total energy intake was fairly constant across age groups (see supplementay data file 1). Mean beverage sugar intake of users was not statistically different between age groups (one-way anova, with post-hoc Bonferroni). Protein from mothers milk is included in animal protein, but contributes a mean of about 10% to total animal protein in the three younger groups and only about 2, 5% in the two older age groups. Formula milk contributes a mean of 47% (sd 37) to animal protein intake. Interactions between beverage sugar and animal protein intake and age, gender, socio-economic status, and maternal overweight were tested, and all were found to be not significant.

**Table 1:** Subject characteristics (in percentage or as mean ± SD)

	Total group (n = 120)			Subgroup (n = 63)		
	Not overweight N = 100	Overweight N = 20	P	Not overweight N = 52	Overweight N = 11	P
Infant age, months	8.5 ± 2.9	9.4 ± 2.6	0.18	8.3 ± 2.9	9.6 ± 2.3	0.21
Infant weight, kg	8.4 ± 1.5	9.0 ± 1.3	0.10	8.2 ± 1.4	9.2 ± 0.9	0.03
Gender, % boys	50	70	0.10	50	73	0.17
High socio-economic status, %	31	35	0.73	33	36	0.81
Breastfed at time of intake assessment, %	21	25	0.69	21	27	0.66
Maternal overweight, %	-	-		39	46	0.67
BMI mother, kg/m^2^	-	-		25.4 ± 5.1	25.6 ± 2.6	0.94
Physical activity, h/week	-	-		14.4 ± 5.3	12.1 ± 5.8	0.20
Duration of breastfeeding, weeks	-	-		35 ± 36	25 ± 24	0.32

**Table 2:** Food, energy and nutrient intake of infants who became overweight or not at 8 years

	Not overweight		Overweight		Difference: overweight vs not overweight			
	n = 100		n = 20					
	Mean	sd	Mean	sd	Mean	95% CI		P
Formula, g	487	325	318	325	-169	-327	-12	**0.04**
Mothers milk^1^, g	85	195	122	243	37	-62	135	0.46
Drinks^2^, g	73	92	162	165	89	11	169	**0.03**
Milk and milk products^2^, g	117	213	181	246	64	-42	170	0.23
Meat, meat products and fowl^2^, g	10.9	16.2	14.5	20.8	3.6	-4.6	11.9	0.39
Energy, kcal	836	202	859	175	23	-73	119	0.64
Energy, kcal/kg	99	18	96	17	-3	-12	5	0.38
Protein, g	26.4	12.4	29.9	12.5	3.5	-2.5	9.5	0.25
Protein, g/kg	3.0	1.1	3.2	1.0	0.2	-0.3	0.7	0.46
Protein, animal^3^, g	19.3	8.7	21.7	9.0	2.4	-1.8	6.6	0.26
Protein, animal, en%	9.0	2.8	9.9	3.0	0.9	-0.4	2.3	0.19
Protein, from formula only, g	8.4	5.5	5.6	5.6	-2.8	-5.5	-0.1	**0.04**
Protein, from formula only, en%	4.2	3.0	2.8	3.0	-1.4	-2.9	0.1	0.06
Protein, animal, complementary foods, g	11.0	11.7	15.8	12.8	4.8	-0.9	10.6	0.10
Protein, animal, complementary foods^4^, en%	4.6	4.7	6.9	5.2	2.3	0.0	4.6	**0.05**
Fat, g	30.4	6.5	30.6	8.6	0.2	-3.2	3.5	0.93
Fat, en%	33.8	7.4	32.6	8.4	-1.2	-4.9	2.4	0.50
Carbohydrate, g	114	33	116	27	2	-14	17	0.82
Carbohydrate, en%	54.1	5.2	53.8	6.5	-0.3	-2.9	2.4	0.86
Sugar, g	74.0	18.2	77.4	18.5	3.4	-5.4	12.2	0.45
Sugar, en%	36.1	7.8	36.3	7.0	0.2	-3.5	3.9	0.91
Beverage sugar^5^, g	10.6	13.9	19.7	20.2	9.1	-0.7	18.9	0.07
Beverage sugar, en%	4.5	5.6	8.8	8.5	4.3	0.2	8.4	**0.04**
								

^1 ^Mothers milk in gram is assessed by frequency and duration of breastfeeding, not weighing.

^2 ^These food groups are predefined by the Dutch Food Composition Table.

3 Protein not from animal sources is predefined by the Dutch Food Composition Table, protein from animal sources is derived by difference between protein and protein not from animal sources.

^4 ^Complementary foods with protein from animal sources are a sum of 6 predefined food groups from the Dutch Food Composition Table: milk and milk products, cheese, eggs, meat/meat products and fowl, fish, and precomposed foods (like meals in a jar; this last group may also contain some protein not from animal sources).

^5 ^For Drinks, Beverage sugar in gram and Beverage sugar in en% were equal variances not assumed, resulting in a non significant differene for Beverage sugar in gram.

A total of 20 out of 120 children (17%) were considered overweight (BMIsds > +1.0) and in the subsample 9 out of 63 children were overweight (14%), which is not significantly different (p = 0.675). Table 3 shows that the unadjusted odds ratio (95% CI) was 1.10 (1.02, 1.18) for beverage sugar intake, and 4.06 (1.50, 11.00) for the highest tertile of animal protein intake compared to the lowest two tertiles. Adjusted odds ratios (model 3) were 1.13 (1.03, 1.24) for beverage sugar and 9.67 (2.56, 36.53) for animal protein, thus both were still independently associated with overweight at 8 years. In the subgroup analysis (n = 63) model 4 showed that after addition of current maternal overweight, full breastfeeding for more than two months, current physical activity in hours per week, and current energy intake in kcal per day, the ORs for beverage sugar and animal protein were still significantly associated with overweight at 8 years.

**Table 3:** Odd ratios for effect of beverage sugar intake and animal protein intake on overweight at 8 years

	Total group N = 120		Subgroup N = 63	
	OR (95% CI)	P	OR (95% CI)	P
Model 1a beverage sugar	1.10 (1.02, 1.18)	**0.009**	1.13 (1.03, 1.25)	**0.014**
Model 1b animal protein	4.06 (1.50, 11.00)	**0.006**	3.32 (0.86, 12.76)	0.080
				
Model 2a beverage sugar	1.10 (1.02, 1.20)	**0.021**	1.15 (1.00, 1.34)	0.058
Model 2b animal protein	7.38 (2.17, 25.10)	**0.001**	5.69 (1.03, 31.45)	**0.046**
				
Model 2 beverage sugar	1.11 (1.03, 1.20)	**0.005**	1.14 (1.03, 1.26)	**0.011**
Model 2 animal protein	4.78 (1.65, 13.87)	**0.004**	3.94 (0.90, 17.27)	0.069
				
Model 3 beverage sugar	1.13 (1.03, 1.24)	**0.009**	1.16 (1.01, 1.33)	**0.042**
Model 3 animal protein	9.67 (2.56, 36.53)	**0.001**	6.77 (1.09, 42.22)	**0.041**
				
Model 4 beverage sugar	-	-	1.21 (1.02, 1.42)	**0.027**
Model 4 animal protein	-	-	11.01 (1.16, 104.2)	**0.036**

Model 1a, 1b: unadjusted effects (a and b indicate two separate analyses)

Model 2a, 2b: adjusted for sex, infant age, infant body weight, breastfed at time of assessment, SES (a and b indicate two separate analyses)

Model 2: adjusted for each other (beverage sugar and animal protein)

Model 3: adjusted for each other, plus sex, infant age, infant body weight, breastfed at time of assessment, SES

Model 4: adjusted as model 3 plus maternal overweight, physical activity in hours per week, and energy intake at 8 years in kcal per day.

When protein from mothers milk was excluded from animal protein, this did not change the OR's. The OR's for becoming overweight for animal protein become slightly lower but demonstrate very significant independent effects (beverage sugar: model 2, OR 1.11 (1.03, 1.19) p = 0.006; model 3, 1.12 (1.02, 1.22) p = 0.014; animal protein: model 2, 3.27 (1.16, 9.19) p = 0.025; model 3, 4.66 (1.47, 14.8) p = 0.009).

**Figure 1:**
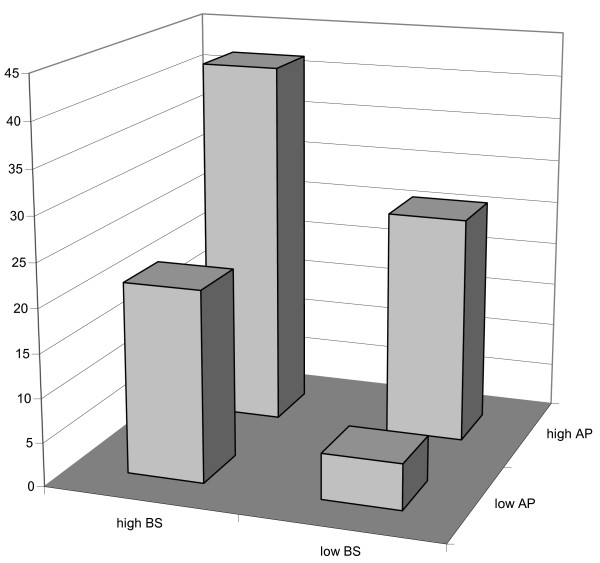
**The prevalence of overweight (%) according to the highest and combined lower tertiles of beverage sugar (BS) intake as well as for animal protein (AP) intake**.

Figure 1 shows that for infants in the lower two tertiles for both beverage sugar and animal protein, only 3 out of 58 infants (5%) were categorised as overweight. Infants in the highest tertile for either beverage sugar or animal protein had 5/23 (22%) or 7/27 (26%) infants with overweight at 8 years, respectively. Infants that were in the highest tertile for both beverage sugar and animal protein had 5/12 (42%) overweight at 8 years. The percentage overweight between the four groups of Figure 1 was significantly different (Chi square test, p = 0.005). Table 4 shows that linear regression analysis provided an effect size of beverage sugar on BMIsds of 0.05 per energy percent of increase in beverage sugar intake, which was not altered by adjustment for confounders. A change in beverage sugar intake of 10 percent of energy intake, might therefore be responsible for an increase in BMIsds of 0.5. For the highest tertile of animal protein intake the increase was 0.5-1.0 BMIsds compared to the lower two tertiles.

**Table 4:** Beta values for effect of beverage sugar intake and animal protein intake on BMIsds at 8 years

	Total group N = 120		Subgroup N = 63	
	Beta (95% CI)	P	Beta (95% CI)	P
Model 1a beverage sugar	0.049 (0.017, 0.081)	**0.003**	0.050 (0.001, 0.099)	**0.047**
Model 1b animal protein	0.47 (0.03, 0.92)	**0.037**	0.69 (-0.01, 1.37)	0.051
				
Model 2a beverage sugar	0.043 (0.006, 0.079)	**0.023**	0.044 (-0.017, 0.104)	0.152
Model 2b animal protein	0.57 (0.11, 1.02)	**0.015**	0.82 (0.12, 1.52)	**0.022**
				
Model 2 beverage sugar	0.049 (0.017, 0.081)	**0.003**	0.049 (0.001, 0.097)	**0.045**
Model 2 animal protein	0.48 (0.05, 0.91)	**0.029**	0.68 (0.01, 1.35)	**0.049**
				
Model 3 beverage sugar	0.044 (0.008, 0.080)	**0.016**	0.049 (-0.009, 0.106)	0.097
Model 3 animal protein	0.59 (0.14, 1.03)	**0.011**	0.86 (0.17, 1.55)	**0.015**
				
Model 4 beverage sugar	-	-	0.055 (-0.005, 0.115)	0.070
Model 4 animal protein	-	-	0.97 (0.23, 1.71)	**0.011**

Model 1a, 1b: unadjusted effects (a and b indicate two separate analyses)

Model 2a, 2b: adjusted for sex, infant age, infant body weight, breastfed at time of assessment, SES (a and b indicate two separate analyses)

Model 2: adjusted for each other (beverage sugar and animal protein)

Model 3: adjusted for each other, plus sex, infant age, infant body weight, breastfed at time of assessment, SES

Model 4: adjusted as model 3 plus maternal overweight, physical activity in hours per week, and energy intake at 8 years in kcal per day.

## Discussion

To our knowledge this is the first study to show two independent effects of both beverage sugar and animal protein intake during infancy and overweight at age 8 years. Beverage sugar intake during infancy increases the risk of overweight at 8 years by more than 10% extra risk per energy percent beverage sugar increase. For animal protein infants with the highest tertile of intake had a more than 9 times higher risk of becoming overweight at 8 years.

In 1995 Rolland-Cachera suggested the early protein hypothesis; a high protein intake in excess of metabolic requirements enhances weight gain in infancy and increases the risk of obesity later in life [5]. This hypothesis has been confirmed in similar small studies [6-8], although not in hispanic youth [9]. The period of dietary exposure, the timing of overweight assessment and the definition of overweight might in part explain different outcomes. Gunther et al. suggested that particularly animal protein might be responsible for the association [27]. When the present analysis was performed with two dummies for second and third tertile of animal protein intake (data not shown), the second tertile did not show a significant relationship. In line with this observation is the hypothesis that animal protein above a certain level of intake might deregulate insulin and IGF-1 with an impact on preadipocyte differentiation and multiplication [2,27]. High protein intakes may also decrease human growth hormone secretion and reduce lipolysis, which might aid fat accumulation [2]. Lower protein levels in infant formula seems to reduce weight up to age 2 years [28], and might therefore contribute to reduction of later overweight.

A recent review by Gibson [14] showed an overwhelming amount of studies on sugar sweetened soft drinks and obesity. Gibson included all cold beverages containing added sugars, carbonated or not. The evidence seems to be inconclusive, although a small effect of sugar sweetened soft drinks on BMI is suggested. A recent study by Herbst et al. [19] is the single study that prospectively investigatedinfant beverage sugar intake during the first year of life. Herbst et al. [19] show no or possibly a protective effect of sugar from beverages and sweets, however the level of beverage sugar intake is very low. At 1 year of age the beverage sugar intake is 0 percent of total energy intake in the Donald study [19] compared to more than 5 percent in the present study. A reasonable explanation for this difference is that the Donald study has selected infants from a higher socio-economic level: > 80% of infants was breastfed for more than 4 months versus 41% in our study, > 60% of parents had a high socio-economic status versus one third in our study, and maternal overweight was only 25% versus > 40% in our study). In line with our study, the authors conclude that when added sugar intake increases to higher levels this might be detrimental to BMI development.

Beverage sugar was consumed through apple and orange juice, most often from apple concentrate, and lemonade also from sugar concentrate. Apple juice has a high fructose content. Fructose consumption results in decreased circulating levels of insulin and leptin when compared with glucose [13]. Because insulin and leptin function as key signals to the central nervous system in the regulation of energy balance, it has been assumed that over a longer period of time high fructose intake could lead to increased caloric intake or decreased caloric expenditure and weight gain [15]. However, at this moment and from this study it is not possible to draw any firm conclusions about the mechanism involved.

Limitations of the study are the total number of infants, although similar studies were of similar size [6-8]. The low response rate to the initial study is also a limitation, but each child serves as its own control. Confounders were not all available for the whole sample of 120 children, but for the subsample of 63. However, the primary effect of beverage sugar and animal protein did not seem to change when tested in the subsample (from model 3 to model 4). Therefore we assume that the effects we found are very robust and would not be different when the whole sample would have provided all information on confounders. Although a large random sample was the basis of the study, socio-economic status (maternal education) was slightly higher compared to Dutch average http://statline.cbs.nl/statweb/. The educational level of parents was very similar to the only other Dutch investigation into dietary intake of infants, the Nutrient Intake Research (VIO) study [29]. This study used a representative sample of 300 infants for both the 9 and the 12 month old infants. We have evaluated the quality of the self reported dietary intake data in an earlier study as good [21], also based on the similarity with the VIO study. Two day food records at one time point is a limited assessment of longer-term exposure to food constituents. However, providing high levels of beverage sugar and/or food products high in animal protein are most likely related to long established food habits of (primarily) the mother. It should also be stressed that both beverage sugar and animal protein were not related to poor food habits. Beverage sugar is largely from fruit juices and from apple concentrate, which the parents judge as 'fruit' and therefore as 'good'. Animal protein is derived from normal healthy food groups and infant formula. The level of intake of percentage energy beverage sugar or animal protein is not significantly different between high and low socio-economic status groups. The relationship between high animal protein intake (highest tertile) with overweight at 8 years is even stronger in high compared to low socio-economic status group (p = 0.006 vs p = 0.102 respectively). This may have resulted in low underreporting as well as high consistency in food 'habits'. Comparing responders with non-responders, no differences appeared in socio-economic status, but parents with breastfed infants at the time of investigation in 2001 had responded to the follow-up call in 2009 significantly more often (26/38 vs 93/188, p = 0.009). Information on maternal smoking is missing, however it is not likely that smoking is associated with beverage sugar or animal protein intake at infancy.

Also, the intake of mother's milk may be more inaccurately estimated than the intake of formula milk.

However, considering the low contribution of mother's milk to the total consumption, it is unlikely to play a key role in effect size and/or significance.

The use of BMIsds as definition of overweight is not ideal but acceptable as an outcome measure; no definition of obesity is ideal at present [16]. Both weight and height are self reported which might introduce some bias. However, recently it has been shown that BMI misclassification was almost absent in an adult population when self reported weight and height were evaluated [30]. Children are more often subject to measurement of weight and height, therefore BMI misclassification is assumed to be limited. Although some BMI misclassification may have occurred it is not likely that the amount of BMI misclassification is related to protein and beverage sugar intake in infancy. It is unlikely this would have caused the significant effect found in this study since the effect of non-differential misclassification on estimates of relative risk will be to always reduce the true relative risk [31]. Physical activity is also self reported, however self reporting is probably less important than selection of a method with a high internal and external validity [32]. Another limitation is the observational nature of the design, which makes it diffecult to conclude a causal nature [2].

Provision of sugar containing beverages and a high animal protein diet in the first year of life appears to increase the risk of overweight at 8 years. Although childhood overweight is clearly multi-factorial, each significant and clinically meaningfull contributing factor that can be influenced by the parents should be addressed accordingly. Sugar containing beverages provided during infancy are much less difficult to address than lifestyle changes during later stages of life [32,33].

Future studies with a larger sample size should confirm these observations.

## Competing interests

The authors declare that they have no competing interests.

## Authors' contributions

PW designed the study, LK, NvB, PW conducted literature research, PW, LK, NvB, SvdZ took part in food calculations and statistical analysis, all authors contributed significantly to writing of the manuscript and all authors read and approved the final manuscript.

## Supplementary Material

Additional file 1 **Table S1. Dietary intake at infancy per age group**. Dietary intake for beverage sugar and animal protein at infancy per age group. Click here for file
