# Supplementary material for: High beverage sugar as well as high animal protein intake at infancy may increase overweight risk at 8 years: a prospective longitudinal pilot study
Source: Nutr J. 2011 Sep 23;10:95. doi: 10.1186/1475-2891-10-95 (PMC3189101; doi:10.1186/1475-2891-10-95)
Supplement: Additional file 1 — Table S1. Dietary intake at infancy per age group. Dietary intake for beverage sugar and animal protein at infancy per age group. [file 1475-2891-10-95-S1.DOC]

Table S1. Dietary intake at infancy per age group.

|  | 4-6m  (n=27) | | 6-8m  (n=27) | | 8-10m  (n=20) | | 10-12m  (n=25) | | 12-13m  (n=21) | | Total  (n=120) | |
| --- | --- | --- | --- | --- | --- | --- | --- | --- | --- | --- | --- | --- |
|  | Mean | SD | Mean | SD | Mean | SD | Mean | SD | Mean | SD | Mean | SD |
| Energy, kcal | 641 | 120 | 767 | 126 | 864 | 128 | 948 | 153 | 1036 | 180 | 840 | 197 |
| Energy, kcal/kg | 96 | 19 | 98 | 17 | 98 | 17 | 98 | 16 | 104 | 19 | 99 | 17 |
| Protein, g | 13.6 | 3.6 | 20.8 | 5.9 | 29.7 | 9.5 | 32.9 | 8.1 | 42.3 | 10.1 | 27.0 | 12.4 |
| Protein, g/kg | 2.0 | 0.6 | 2.7 | 0.7 | 3.4 | 1.1 | 3.4 | 0.8 | 4.2 | 1.0 | 3.1 | 1.1 |
| Protein, en% | 8.4 | 1.2 | 10.6 | 1.8 | 13.8 | 4.2 | 13.8 | 2.3 | 16.4 | 3.1 | 12.3 | 3.8 |
| Protein non-animal, en% | 0.8 | 0.7 | 2.6 | 1.1 | 3.7 | 1.2 | 4.4 | 0.9 | 5.0 | 1.1 | 3.2 | 1.8 |
| Protein animal, en% | 7.6 | 1.5 | 8.0 | 1.7 | 10.0 | 3.9 | 9.4 | 1.9 | 11.5 | 3.3 | 9.1 | 2.8 |
| Protein animal for highest tertile, en% | 9.3 | 0.9 | 9.8 | 0.7 | 13.4 | 4.9 | 11.2 | 1.0 | 15.1 | 0.8 | 11.6 | 3.0 |
| Protein animal (excl.mothers milk), en% | 6.9 | 2.9 | 7.2 | 3.0 | 9.2 | 4.8 | 9.2 | 2.3 | 11.2 | 3.5 | 8.6 | 3.6 |
| Carbohydrates, en% | 49.7 | 5.4 | 55.7 | 4.4 | 53.4 | 4.3 | 57.0 | 3.8 | 54.5 | 6.0 | 54.0 | 5.4 |
| Fat, en% | 41.9 | 5.5 | 33.6 | 5.2 | 32.8 | 7.5 | 29.2 | 4.6 | 29.0 | 6.9 | 33.6 | 7.6 |
| Sugar, g | 62.9 | 20.3 | 74.8 | 17.8 | 76.9 | 12.2 | 80.5 | 15.5 | 79.9 | 18.1 | 74.5 | 18.2 |
| Beverage sugar, g | 2.2 | 5.5 | 6.7 | 11.5 | 11.7 | 11.2 | 20.8 | 16.8 | 22.0 | 19.5 | 12.1 | 15.4 |
| Sugar, en% | 39.4 | 10.4 | 39.2 | 7.8 | 35.8 | 4.1 | 34.0 | 4.1 | 31.0 | 5.2 | 36.2 | 7.6 |
| Beverage sugar, en% | 1.3 | 3.2 | 3.3 | 5.3 | 5.4 | 5.3 | 8.7 | 7.0 | 8.5 | 7.1 | 5.2 | 6.3 |
| Beverage sugar for highest tertile, en% | 8.8 | 1.0 | 8.9 | 6.1 | 11.2 | 3.6 | 16.6 | 6.6 | 16.7 | 4.7 | 12.7 | 6.1 |
| Beverage sugar (users only), en% | 8.8 | 1.0 | 6.9 | 5.9 | 7.7 | 4.6 | 9.4 | 6.8 | 10.0 | 6.7 | 8.7 | 6.0 |
| Users of beverage sugar, % infants | 14.8 |  | 48.1 |  | 70.0 |  | 92.0 |  | 85.7 |  | 60.0 |  |

En%, percentage of total energy intake
